# Two years of ethics reflection groups about coercion in psychiatry. Measuring variation within employees’ normative attitudes, user involvement and the handling of disagreement

**DOI:** 10.1186/s12910-023-00909-w

**Published:** 2023-05-12

**Authors:** Bert Molewijk, Reidar Pedersen, Almar Kok, Reidun Førde, Olaf Aasland

**Affiliations:** 1 Centre for Medical Ethics, Institute of Health and Society, University of Oslo, Oslo, Norway; 2 Department of Ethics, Law and Humanities, Amsterdam UMC, Location VUmc, Vrije Universiteit Amsterdam, Amsterdam, The Netherlands; 3 Department of Epidemiology and Data Science and Department of Psychiatry, Amsterdam UMC Location Vrije Universiteit Amsterdam, De Boelelaan 1117, Amsterdam, The Netherlands; 4 Aging and Later Life, Amsterdam Public Health Institute, Amsterdam, The Netherlands

**Keywords:** Ethics reflection groups, Moral case deliberation, Coercion, Attitudes, Clinical ethics support, User involvement, Constructive disagreement, Mental health care, Outcomes evaluation, Repeated cross-sectional survey

## Abstract

**Background:**

Research on the impact of ethics reflection groups (ERG) (also called moral case deliberations (MCD)) is complex and scarce. Within a larger study, two years of ERG sessions have been used as an intervention to stimulate ethical reflection about the use of coercive measures. We studied changes in: employees’ attitudes regarding the use of coercion, team competence, user involvement, team cooperation and the handling of disagreement in teams.

**Methods:**

We used panel data in a longitudinal design study to measure variation in survey scores from multidisciplinary employees from seven departments within three Norwegian mental health care institutions at three time points (T0–T1–T2). Mixed models were used to account for dependence of data in persons who participated more than once.

**Results:**

In total, 1068 surveys (from 817 employees who did and did not participate in ERG) were included in the analyses. Of these, 7.6% (N = 62) responded at three points in time, 15.5% (N = 127) at two points, and 76.8% (N = 628) once. On average, over time, respondents who participated in ERG viewed coercion more strongly as offending (*p* < 0.05). Those who presented a case in the ERG sessions showed lower scores on User Involvement (*p* < 0.001), Team Cooperation (*p* < 0.01) and Constructive Disagreement (*p* < 0.01). We observed significant differences in outcomes between individuals from different departments, as well as between different professions. Initial significant changes due to frequency of participation in ERG and case presentation in ERG did not remain statistically significant after adjustment for Departments and Professions. Differences were generally small in absolute terms, possibly due to the low amount of longitudinal data.

**Conclusions:**

This study measured specific intervention-related outcome parameters for describing the impact of clinical ethics support (CES). Structural implementation of ERGs or MCDs seems to contribute to employees reporting a more critical attitude towards coercion. Ethics support is a complex intervention and studying changes over time is complex in itself. Several recommendations for strengthening the outcomes of future CES evaluation studies are discussed. CES evaluation studies are important, since—despite the intrinsic value of participating in ERG or MCD—CES inherently aims, and should aim, at improving clinical practices.

**Supplementary Information:**

The online version contains supplementary material available at 10.1186/s12910-023-00909-w.

## Background

In their continuous aiming for quality of care, health care professionals inherently experience various kinds of moral challenges. Health care professionals report that dealing with these moral challenges in a methodologically sound and constructive way is often difficult [1–8]. To support health care professionals in dealing more systematically with moral challenges, different types of clinical ethics support (CES)—such as ethics consultants, clinical ethics committees and moral case deliberations (MCD) or ethics reflection groups (ERG)^1^—have been developed [9–13]. Many papers on CES implicitly or explicitly state that CES not only supports professionals with respect to the handling of the specific case at hand, but also contributes to the moral competency of professionals, multidisciplinary team cooperation and, in the end, a better quality of care [14–17]. Although most participants in CES repeatedly report satisfaction with the ethics support, there is still little research on possible outcomes of CES, nor research that focuses specifically on the impact of CES on clinical practice and quality of care. In particular, there is a lack of research that measures changes in relevant outcomes of CES. In this paper, we present the results of a study in which we report variation in outcomes during three time points after implementing regular ERG sessions over 24 months at seven departments in three Norwegian institutions for mental health care. All sessions dealt with employees’ moral challenges related to the use of various coercive measures.

### Evaluation of clinical ethics support

CES evaluation studies are of crucial importance, since they may contribute to the further development of the relatively young professional domain of CES. Executing and reporting CES evaluation research can be seen as a way of exchanging lessons learned, offering input for (developing) training for CES staff, and evoking critical questions about justification, appropriateness, method, quality, and impact of CES. Regarding the impact of CES, both critics and advocates of CES state that evaluation research focusing on the impact of CES is needed to clarify the usefulness of CES [30–35]. However, measuring the impact of CES is complex. CES, and ERG in particular, can easily be understood as a complex intervention, the ingredients of which are often unclear or not made explicit [36–39]. There is a variety of different ingredients of ERG (e.g. the training of the ERG facilitators, the specific context in which the ERG is implemented, the conversation method used within ERG, the motivation and inquisitiveness of the ERG participants, and the characteristics of the case at hand). Furthermore, CES evaluation (review) studies focus on a variety of different issues, such as structure, process, content, outcomes and efficiency of CES [18–29].^2^

Indeed, high-quality prospective CES evaluation studies which include baseline and follow-up measurements are rare [23, 25, 40–42]. A recent Cochrane review, studying the available evidence of controlled studies of the effectiveness of ethical case interventions for adult patients, included 6 articles from 4 randomised trials [43]. It concluded that it was not possible to determine the effectiveness of CES due to low quality of the evidence presented in those studies. The authors end with a plea for future research to identify and measure CES-related outcomes, taking into account the different goals of different types of CES interventions.^3^ Yet not all CES outcomes are equally important, feasible or even desirable and should therefore not automatically become the aims and justification of CES.^4^ Hence, when looking for variation in CES outcomes over time, it is important to focus on the right kind of CES outcomes, which match the specific goals of the CES, the specific CES intervention, and the specific context in which CES is implemented [35, 43].

### Outcomes tied to specific CES intervention: Ethics Reflection Groups

Regarding studies describing or evaluating outcomes for ERG or MCD, some qualitative and self-reported evaluation studies indicate that MCD and ERG sessions can lead to improved team cooperation [10, 18, 19, 21, 51–53]. This fits well within the results of a recent systematic review on the impact of MCD, covering 25 empirical evaluation papers: MCD participants reported that MCD can bring about improvements in inter-professional interactions [23]. Furthermore, given the specific characteristics of MCD and ERG (i.e. learning from different viewpoints in constructive and respectful dialogues and putting yourself in someone else's shoes), qualitative evaluation studies reported that MCD and ERG contributed to a more constructive handling of disagreement in teams [4, 54]. Finally, as MCD and ERG include elucidation of the values and norms of patients and their family, and moral challenges from their perspectives as well, it has been suggested that MCD and ERG contribute to a better understanding of the viewpoints of patients and next of kin [10, 20, 21, 55, 56].

### Outcomes tied to the specific context: Changing staff attitudes regarding the use of coercion

In mental health care the use of coercion is one of the most pressing ethical issues, and many qualitative studies report negative experiences of patients exposed to coercion [57]. At the same time, quantitative research on the relationship between the use of coercive measures and patient outcomes is sparse [58]. Many express strong criticisms of the use of coercion in mental health care, while others argue that limited use of coercion is ethically acceptable when the benefits regarding protection or treatment outweigh the negative effects on patients’ autonomy, integrity and comfort [57, 59, 60]. Independent of one’s view on the use of coercion, a critical reflection on the use of coercion (including the timing, duration, alternatives, proportionality and the effectiveness of its use) is always needed, since the use of coercion involves an infringement of patients’ autonomy and integrity. Hence, coercion is, and should be, always an intervention with complex value conflicts. Yet, these value conflicts are often implicit and not explicitly addressed and weighed.

A change in the staff’s normative attitudes regarding the use of coercion, as well as in department culture, may be key to increase critical reflection on the use of coercion, reduce the use of coercion and make the use of coercion more morally appropriate [61–64]. Scanlan [65] writes that training to promote change in attitudes is essential, since without substantial shifts in staff attitudes, efforts to reduce the use of seclusion and restraint are unlikely to be successful [66, 67]. Changing the department culture and staff attitudes is challenging. However, various explorative research projects on the use of coercion indicate that use of ethics reflection, such as MCD and ERG sessions, can contribute to a more critical culture and a more critical attitude towards the use of coercion [54, 68–70]. To our knowledge, quantitative research on how to change normative attitudes towards the use of coercion is scarce.

Our current study focuses on studying the correlation between structural participation in ERGs on the one hand and the change of respondents’ normative attitudes with respect towards the use of coercion on the other hand. In addition, we studied whether respondents report that they involve patients and family more regarding the use of coercion, whether their team cooperation improved and whether they handled disagreements in their teams more constructively.

## Research questions

In this study we looked at the following outcome parameters:

- The employees’ normative attitudes towards the use of coercive measures;
- The way employees report about the factual competence of the team regarding the handling of coercion;
- The way employees report about the factual involvement of patients and families in situations in which coercive measures has been or may be used;
- The way employees think about the quality of the cooperation in their team;
- The way employees perceive the handling of disagreements in their team.

We used the following research questions:

1. Do the seven outcome parameters differ between the following time points
  - T0: before implementation of Ethics Reflection Groups (ERG);
  - T1: 1 year after ERG implementation;
  - T2: 2 years after ERG implementation?
2. Do the seven outcomes differ according to department and profession at the three time points?
3. Do outcomes change within persons over time?

Based on the ERG and MCD evaluation literature, and studies related to changing practices and attitudes regarding the use of coercion, our general hypotheses were as follows:

- ERG participants develop a more critical view on the use of coercive measures;
- ERG participants increase their attention for patient and family involvement in situations concerning the (possible use of) coercion; and
- ERG participants report an improvement in team cooperation and constructive handling of disagreement within the teams.

## Context of the study

The results presented in this paper are part of a larger study called “mental health care, ethics and coercion” (further referred to as “PET”, based on the Norwegian abbreviation for the study: Psykiatri, Etikk & Tvang).^5^ Based on availability and motivation, seven departments from three different mental health care institutions in three different Norwegian counties joined the study. From these departments, 23 employees were trained, during 5 training days, as ERG facilitators by ethicists from the Centre for Medical Ethics (CME) at the University of Oslo.^6^ Usually, two newly trained facilitators facilitated each single ERG session at their own department. For two years, ERGs took place once or twice a month at every department. Multidisciplinary health care professionals (i.e. nurses, socio-therapists, psychologists, psychiatrists, doctors, physiotherapists, quality management staff, team leaders, managers) participated voluntarily in the groups. The ERG sessions lasted between 50 and 90 min; 2 to 20 people participated in each group [72]. A step-by-step ethics reflection model, the CME model, was utilised in the deliberations [6].

Various research methods were utilised to study the implementation and evaluation of ERGs. The survey questionnaire which compiled the data for this paper consisted of several thematic areas. In this paper, we focus on differences in the outcomes between the three time points, whether there are differences between various departments and professions, and—in a subgroup of persons who participated in the surveys two or three times—whether there were associations between ERG participation and changes in the seven outcome parameters over time.

## Method

### Design

A survey was distributed three times among employees from various disciplines working in the same seven departments within three Norwegian mental health care institutions, with one year in between each time point (T0–T1–T2). New participants were allowed to enter the study at follow-up. Most participants (77%) filled out the survey only once, but some employees participated two or three times and therefore provided longitudinal data.

### Study sample

The study sample existed of the employees from the seven participating departments. From hospital 1 a geriatric department was included, from hospital 2 an emergency, a community, and a youth and a specialist care department, and from hospital 3 an emergency and a rehabilitation department were included. During this study, all these departments held regular ERG sessions during a period of two years. The employees consisted of various health care professionals, such as nurses, auxiliary nurses, psychiatrists (including psychiatrists in training), and psychologists, as well as team leaders and management personnel. Employees were invited by the local study coordinator (an employee at their department) and/or management to fill out the written questionnaire either during team or department meetings or individually by email. Temporary staff and supporting staff did not participate in the study.

### Research instruments

The survey used in this paper was distributed before the departments started with ERG sessions. This survey was used as a baseline (T0). This survey was used at 12 months (T1) and at 24 months (T2) after the start of the ERG sessions. The ERGs dealt with ethical challenges related to the use of coercion in concrete situations as experienced by the health care staff. An earlier version of the survey was piloted for clarity by various health care professionals and commented on by members of the PET Sounding Board, who are expert researchers in the field of coercion. The survey contained the following dependent variables, independent variables and co-variates.

#### Dependent variables^7^

##### Staff’s normative attitudes regarding the use of coercion

Staff’s normative attitudes regarding the use of coercion were measured with the validated Staff’s Attitude to Coercion Scale (SACS) [77]. The SACS concerns the use of coercion in general and includes formal, informal and experienced coercion. It consists of 15 normative statements representing three subscales (see Additional file 1: Textbox 1 for the SACS statements):

- Coercion seen as offending (**SACS I; 6 items; ‘offending’**);
- Coercion seen as needed for care and security (**SACS II; 6 items; ‘care & security’**); and
- Coercion seen as treatment (**SACS III; 3 items; ‘treatment’**).

Each item was scored on a Likert scale ranging from 1 (strongly disagree) to 5 (strongly agree). For each subscale we calculated the mean of the items and used these as dependent variables in separate models. Mean scores on ‘Offending’ and ‘Care & security’ were calculated only if respondents had valid answers on at least 4 of the 6 items; for ‘Treatment’ when each of the three items was answered validly.

Textbox 1: The 15 normative statements of the SACS [77].

##### Coercion competence of the team

We developed 6 factual statements^8^ in order to find out how the respondents evaluated the competence of the team in dealing with coercion. The statements were tested for clarity in the same pilot study we mentioned earlier, but they were not validated (see Textbox 2). Each item was scored on a Likert scale ranging from 1 (strongly disagree) to 5 (strongly agree), and a mean score was calculated.

Textbox 2: The 6 statements about the competence of the team regarding use of coercion.

##### Involvement of patients and family in situations of coercion

We developed 11 factual statements in order to find out to which degree respondents thought they involve patients and family before, during and after situations of coercion. The statements were tested for clarity in a pilot study yet not validated (see Textbox 3). Each item was scored on a Likert scale ranging from 1 (never) to 3 (once in a while) to 5 (almost always), and a mean score was calculated.

Textbox 3: The 11 statements about involvement of patients and family in situations of coercion.

##### Team cooperation

We made use of 13 factual statements from two validated questionnaires in order to ask respondents how they thought about the cooperation within their team: 10 items from the Team Reflexivity Scale [78] and 3 items from the Tolerance and Openness Scale [79] (see Textbox 4). The combined statements were tested for clarity in a pilot study, but not validated. Each item was scored on a Likert scale ranging from 1 (strongly disagree) to 5 (strongly agree), and a mean score was calculated.

Textbox 4: The 13 statements about team cooperation.

##### Constructive disagreement

We used 8 statements from the validated Constructive Confrontation Norms questionnaire [80] (see Textbox 5). The statements were tested for clarity in a pilot study, but not validated. Each item was scored on a Likert scale ranging from 1 (strongly disagree) to 5 (strongly agree), and a mean score was calculated.

Textbox 5: The 8 statements about constructive disagreement.

#### Independent variables

##### Participation in ethics reflection groups

At T1 and T2, respondents were asked whether they participated in ERGs in the last 12 months (yes/no) and if yes, how often during the last 12 months (0 times, 1–5 times, 6–12 times, 13 or more times). In the analysis we merged the latter two groups into one: 6 or more times because only a small number of respondents participated in ERGs that often.

##### Presentation of a case in the Ethics Reflection Groups

At T1 and T2, respondents were asked whether they had presented a case in ERGs in the last 12 months (yes/no) and if yes, how often during the last 12 months (0 times, 1 time, 2 to 4 times, more than 4 times). Because only a small number of respondents presented a case often, we merged the latter two groups into one: 2 times or more.

#### Covariates

##### Department

The department that the respondents belonged to was a nominal variable, i.e. participants could indicate only a single department. We dummy-coded all seven departments and added them to the models, except for the Hospital 2 Acute Care dummy. As a result, Hospital 2 Acute Care was the reference group in all analyses.

##### Type of profession

We categorized the respondents’ professions into 5 categories: 1) ‘psychologists’, 2) ‘psychiatrists and related medical professions’ (e.g. psychiatrist in training, physician, chief-physician), 3) ‘nurses & related professions’ (e.g. auxiliary nurses, milieu therapist, helping assistant), 4) ‘management’ (unit team leader, department manager, director), and 5) ‘other professions’ (e.g. physiotherapist, occupational therapist, creative therapist, and other). Temporary staff and supporting staff did not participate in the study. For employees who participated more than once, only their baseline profession was included. ‘Psychiatrists and related medical professions’ were used as the reference group in all analyses.

##### Demographics

Age was categorised into younger than 29, 30–49 years, and 50 years or over. Gender was coded as 1 (female) and 0 (male). Age at the first participation was used in the analyses.

### Analytic strategy

First, we provide descriptive statistics of all variables for each time point separately. Subsequently, because some participants provided multiple, repeated observations, we used linear mixed models in SPSS v22 to take into account the dependency between their observations [81]. This method enabled us to incorporate all available observations, including those from participants with repeated measures, for whom dependency of these repeated measures is considered.

We estimated differences in average outcomes between time points by adding two dummy variables for T1 and T2 to the models, using T0 as reference group. To test whether differences in outcomes between time points differed between departments and professions, we added interaction effects between the two-time dummies and department or profession, respectively. We used the default Restricted Maximum Likelihood (REML) method to estimate the regression coefficients.

We estimated three models. In the first and second model we included the complete sample, where most of the participants provided data on only a single time point. Therefore, in these models we modelled each time point separately by dummy-coding T1 and T2. The effects of T1 and T2 can be interpreted as the difference in the outcome at T1 and T2 compared to T0, respectively. Furthermore, we estimated average differences in the seven outcome parameters between departments and professions, regardless of time. We additionally adjusted for the number of times participants took part in the survey (1, 2, or 3 times).

Model 2 focused on the question whether departments and professions differed in the changes in outcomes over time, using interaction effects with the T1 and T2 time dummies as described above. For establishing differences in outcomes, we needed to interpret two coefficients estimated in the mixed models. First, in model 2, the main effect of the time dummies expresses the difference between that time point and T0 in the reference category of the predictors entered in the interaction effect. For example, for T1 this would be the mean difference in [outcome] between T0 and T1 for Hospital 2 Acute Care. Second, the interaction effect, indicated as Time*[predictor]; this coefficient expresses how much larger or smaller the difference between time points is in the group of interest, compared to the difference in the reference group. By adding the coefficient of the interaction effect to the main effect of time, the difference between time points in the group of interest can be calculated. The p-value of the interaction effect indicates the statistical significance of the difference between the departments or professions in the change in outcomes between two time points. We again emphasize that, given the limited number of participants with longitudinal data, these estimates should be interpreted as differences in department or profession group averages between time points, and not as average changes in outcomes over time on the individual level.

In model 3, we specifically focused on the effects of ERG participation and case presenting on changes in outcomes across time on the individual level. Therefore, we estimated the third model only in participants with at least two observations (N = 160). The model focused on the effects of the number of times participants took part in ERGs (data for N = 160) and the number of times they presented a case (data for N = 109). In this third model, we treated time as a continuous variable because data on change was available for all individuals in the dataset, and because we were interested in any gradual change in outcomes across the entire observation period that might be associated with ERG participation or case presenting. Coefficients of this model can be interpreted as the mean individual change in the outcomes *per year.* To isolate the intervention effect (i.e. the effect of ERG), the model was adjusted for department and profession, and for outcomes observed at baseline.

We used *p* < 0.05 as the cut-off point for statistical significance. Age and gender were found to be unrelated to the outcomes and were therefore not included as covariates in the models.

## Results

In total, 1068 responses from 817 employees (including those who did and did not participate in ERG) were included in the analyses. Of these, 7.6% (N = 62) responded at all three points in time, 15.5% (N = 127) at two points, and 76.8% (N = 628) once. Hence, there are repeated measures for 7.6% + 15.5% = 23.1%. Respondents entered the study at different times.

### Descriptive analyses

An overview of descriptive statistics of the sample is provided in Table 1.

**Table 1:** Descriptive statistics of respondents and overall scores for the 7 scales

	T0		T1			T2		
	Valid n	Count (%) or mean (SD)	Valid n	% or mean (SD)		Valid n		% or mean (SD)
**Demographics**								
*Male gender* (n (%))	390	154 (39.5)	348	122 (35.1)		271		94 (34.7)
*Age groups* (n (%))	389		344			263		
< 29		53 (13.6)		43 (12.5)				34 (12.9)
30–39		118 (30.3)		105 (30.5)				66 (25.1)
40–49		108 (27.8)		96 (27.9)				86 (32.7)
50–59		82 (21.1)		77 (22.4)				52 (19.8)
> 60		28 (7.2)		23 (6.7)				25 (9.5)
**Participation in ERGs** (n (%))	N/a		353			284		
No				172 (48.7)				128 (45.1)
1–5 times				138 (39.1)				108 (38.0)
6 + times				43 (12.1)				48 (16.9)
**Presented an own case in ERGs** (n (%))	N/a		205			158		
No				111 (54.1)				80 (50.6)
1 time				52 (25.4)				45 (28.5)
2 + times				42 (20.5)				33 (20.9)
**Attitudes to coercion** (SACS, 1–5)								
Offending	393	3.11 (0.56)	348	3.10 (0.55)		274		3.13 (0.58)
Care and Security	394	4.11 (0.50)	352	4.01 (0.50)		276		3.99 (0.55)
Treatment	379	2.58 (0.65)	337	2.53 (0.68)		270		2.50 (0.65)
**Coercion team competence** (6 items; 1–5)	383	3.65 (0.55)	334	3.69 (0.68)		262		3.66 (0.61)
**User involvemen**t (11 items; 1–5)	354	2.82 (0.65)	282	2.92 (0.72)		228		3.03 (0.71)
**Team cooperation** (13 items; 1–5)	361	3.70 (0.49)	329	3.75 (0.49)		250		3.72 (0.50)
**Constructive disagreement** (8 items; 1–5)	366	3.57 (0.53)	303	3.61 (0.56)		252		3.60 (0.58)
**Hospital and department type** (n (%))		First reported across T0-T2 (as used in the mixed model analyses)						
*Hospital 1*	**760**							
Geriatric care		71 (9.3)						
*Hospital 2*								
Acute care		187 (18.3)						
Community care		139 (10.7)						
Youth care		81 (10.1)						
Specialist care		77 (14.1)						
*Hospital 3*								
Acute care		107 (12.9)						
Rehabilitation care		187 (24.6)						
**Profession** (n(%))		First reported across T0-T2 (as used in the mixed model analyses)						
	**626**							
Psychologists		63 (10.1)						
Psychia. & related		69 (11.0)						
Management		19 (3.0)						
Nurses & related		314 (50.2)						
Other		161 (25.7)						

Descriptive statistics of all available data (n = 817 subjects)

We observed some differences between T0, T1 and T2 with respect to the average scores for all seven parameters of all respondents together (i.e. those who participated in ERG and those who did not participate in ERG). Regarding respondents’ attitude about coercion, across all three time points, on average respondents did not strongly agree nor strongly disagree with the viewpoint that coercion can be offensive. Care and Security scores varied between 4.11 at T0 and 3.99 at T2, indicating a slight average agreement with justifying the use of coercion for reasons of care and security. Scores on the Treatment scale varied between 2.58 at T0 and 2.50 at T2, indicating a modest average disagreement with the idea that coercion can be seen as a form of treatment. Respondents were on average slightly positive about the current team competence for using or preventing coercion (scores varied between 3.65 at T0 and 3.66 at T2). Regarding user involvement in the prevention, execution and evaluation of coercion, the average score was 2.82 at T0 and 3.03 at T2. On average, respondents slightly agreed that they had good team cooperation (scores ranging from 3.70 at T0 and 3.72 at T2). Finally, on average respondents slightly agreed that they handled disagreement constructively (scores between 3.57 at T0 and 3.61 at T1).^9^

### Mixed model results: general variation at three time points and outcomes on 5 parameters

#### General variation at three time points (see Table 2)

**Table 2:** Adjusted associations between time, survey participation, ERG participation, department and profession

	SACS offending	SACS Care/Security	SACS Treatment	Team Coercion Competence	User Involvement	Team Cooperation	Constructive Disagreement
**Variable**	B adj.^a)^	B adj.^a)^	B adj.^a)^	B adj.^a)^	B adj.^a)^	B adj.^a)^	B adj.^a)^
**Time**							
Intercept(Mean at T0)	3.13	4.11	2.59	3.64	2.81	3.69	3.55
T1 vs T0	−0.02	−0.10*	−0.02	0.06	0.08	0.05	0.05
T2 vs T0	0.02	−0.12**	0.01	0.03	0.17**	0.02	0.08 ~
**Department**	*reference category* = *Hospital 2 Acute*						
Intercept (Mean in ref.)	3.20	4.13	2.64	3.65	2.71	3.73	3.60
Hosp.1Geriatric	−0.11	−0.06	−0.10	−0.19 ~	0.18	−0.24**	−0.30**
Hosp.2Community	−0.01	−0.06	−0.16 ~	0.03	0.19*	0.06	0.04
Youth	−0.18*	−0.15*	−0.26**	−0.17 ~	0.29**	< 0.01	−0.09
Specialist	0.06	−0.19*	−0.14	0.06	0.35**	0.05	0.06
Hosp.3Acute	−0.16*	0.07	0.12	0.20*	0.32***	0.04	−0.10
Rehabilitation	−0.32***	0.07	0.01	0.16 ~	0.13	0.09	0.10
**Profession**	*reference category* = *Psychiatrists and related professions*						
Intercept (Mean in ref.)	3.12	4.06	2.47	3.70	3.18	3.79	3.71
Psychologists	0.44***	−0.22*	−0.18	−0.06	−0.04	−0.23*	−0.13
Nurses	0.06	< 0.01	0.01	−0.08	−0.32***	−0.04	−0.12
Other professions	0.03	−0.11	0.08	−0.07	−0.24*	−0.10	−0.18*
Management	−0.34*	0.12	−0.04	0.24	−0.30 ~	< 0.01	0.05

a) Adj. = Coefficients adjusted for time, department, profession, and number of times responded. Intercepts are unadjusted

~ *p* < 0.1; *p < 0.05; **p < 0.01; ***p < 0.001

b) We executed additional analyses in which we adjusted for number of times that participants were included in the survey. We found 6 significant differences (*p* < 0.05) and therefore we adjusted for numbers of times that participants were included in the survey

c) ERG Participation and Case Presenting are removed from this analysis towards a separate analysis that is restricted to persons who participated at least twice and had baseline data on the outcomes (see part IV and Table 4 in this Results section)

Adjusted associations between time, department, profession and general levels of seven outcomes; Based on linear mixed models (additionally adjusted for number of times participants were included in the survey^b^)^c^

On average, **SACS Care & Security** was *b* = 0.10 lower at T1 and *b* = 0.12 lower at T2 than at T0 (*p* < 0.05 and *p* < 0.01, respectively). The average was 4.11 at T0, 4.01 at T1 and 3.99 at T2, indicating that at later time points, participants agreed slightly less that coercion is a form of care or security. Furthermore, **User Involvement** was on average 2.98 at T2 while it was 2.81 at T0 (*p* < 0.01), indicating that at T2, participants thought they involved patients and their family significantly more often in situations of coercion. No other statistically significant differences in average outcomes between the time points were found.

#### Differences and similarities in outcomes between Departments and Professions

We observed significant differences in the outcomes for the 5 parameters between Departments and Professions. For example, compared to the Hospital 2 Acute Department (reference), the Hospital 3 Rehabilitation Department scored on average 0.32 lower on **Offending** (*p* < 0.001), indicating that employees from the Rehabilitation Departments perceived coercion as less offending than employees from the Acute Department (Hospital 2). With respect to **User Involvement**, the Hospital 2 Specialist Department and the Hospital 3 Acute Department scored 0.35 (*p* < 0.01) and 0.32 (*p* < 0.001) higher than the reference department, respectively; i.e. they involved patients and family in situations of coercion more often. The Hospital 1 Geriatric Department score 0.30 lower on **Constructive Disagreement** (*p* < 0.01); i.e. it perceived the way of dealing with disagreements within the team as less constructive.

Furthermore, compared to the category ‘psychiatrists and related medical professions’ (i.e. the reference group), psychologists experienced coercion more strongly as **Offending** (b = 0.44, *p* < 0.001), less as a form of **Care & Security** (b = −0.22, p < 0.05), and perceived less **Team Cooperation** (b = −0.23, *p* < 0.05). Managers perceived coercion less strongly as **Offending** (b = −0.34, *p* < 0.05) than ‘psychiatrists and related medical professions’. Finally, nurses perceived substantially less **User Involvement** than ‘psychiatrists and related medical professions’ (b = −0.32, *p* < 0.001).

#### Adjustments based on number of times participants were included in the survey

We performed additional analyses in which we adjusted for the number of times participants were included in the survey. We found 6 significant differences (*p* < 0.05) and concluded that we needed to adjust the initial analyses. However, differences in the results were small. Generally, those who participated more often in the study tended to see coercion less strongly as Offending and less as a form of Care & Security. They also score higher on Team Coercion Competence and User Involvement.

### Differences between time points for departments and professions

We will now present differences in outcomes associated with Departments and Professions between the three time points (see Table 3). Interactions between time and Department and Profession were calculated in two separate models.

**Table 3:** Interaction effects between time (T1, T2) and department and profession

	SACS offending	SACS Care/Security	SACS Treatment	Team Coercion Competence	User Involvement	Team Cooperation	Constructive Disagreement
**Variable**	B adj.^a)^	B adj	B adj	B adj	B adj	B adj	B adj
**Department**	*reference category* = *Hospital 2 Acute*						
Intercept(T0 mean ref)	3.13	4.22	2.60	3.73	2.91	3.78	3.73
Main effect t1 vs t0	0.002	−0.09	0.03	−0.07	0.05	−0.01	0.02
Main effect t2 vs t0	0.17*	−0.09	−0.01	−0.06	0.24*	−0.06	−0.06
T1*Department^b)^							
Hosp.1Geriatric	0.02	−0.001	−0.34 ~	0.11	0.24	0.003	−0.19
Hosp.2Community	−0.15	−0.02	0.23	0.07	−0.02	−0.02	−0.10
Youth	0.03	−0.06	−0.01	0.05	−0.22	0.15	0.23 ~
Specialist	−0.07	0.01	−0.03	0.62**	0.38	−0.11	0.10
Hosp.3Acute	−0.01	−0.02	−0.07	0.14	0.03	0.06	0.03
Rehabilitation	−0.15	0.12	−0.35 ~	0.35*	0.20	0.42**	0.19
T2*Department							
Hosp.1Geriatric	−0.18	−0.08	−0.15	−0.01	−0.20	−0.13	−0.02
Hosp.2Community	−0.41**	−0.03	0.29 ~	0.09	0.03	0.17	0.11
Youth	−0.01	−0.10	−0.01	0.02	−0.11	0.23 ~	0.37**
Specialist	−0.14	−0.10	−0.05	0.10	0.51 ~	−0.09	0.13
Hosp.3Acute	−0.12	0.04	0.002	0.22	−0.32 ~	0.05	0.09
Rehabilitation	−0.36 ~	−0.05	−0.18	0.30	0.05	0.25	0.25
	SACS offending	SACS Care/Security	SACS Treatment	Team Coercion Competence	User Involvement	Team Cooperation	Constructive Disagreement
**Profession**	*reference category* = *Psychiatrists and related professions*						
Intercept	3.21	4.25	2.59	3.60	2.77	3.73	3.66
Main effect t1 vs t0	−0.06	−0.17	−0.03	0.13	0.31*	−0.01	0.02
Main effect t2 vs t0	−0.05	−0.05	0.13	0.11	0.31 ~	0.21 ~	0.25*
T1*Profession^b)^							
Psychologists	0.07	0.01	0.06	0.14	−0.09	0.05	0.02
Nurses	0.02	0.08	0.09	−0.11	−0.22	0.06	0.01
Other professions	0.003	0.15	−0.004	−0.11	−0.07	0.08	0.07
Management	0.42 ~	−0.15	−0.84**	0.05	0.24	0.23	0.19
T2*Profession^b)^							
Psychologists	0.29	−0.28	−0.23	0.06	0.11	−0.02	0.13
Nurses	0.05	−0.06	−0.07	−0.15	−0.18	−0.26 ~	−0.21
Other professions	0.08	−0.05	−0.18	−0.05	−0.06	−0.25 ~	−0.27 ~
Management	−0.09	−0.24	−0.75*	0.17	−0.46	0.24	0.18

a) Adj. = Adjusted for survey participation, department and profession

b) Interpretation: difference in yearly change compared to the change in the reference group

~ *p* < 0.1; *p < 0.05; **p < 0.01; ***p < 0.001

#### *Departments* (with Acute Care from Hospital 2 functioning as reference group; see Fig. 1)

**Fig. 1:**
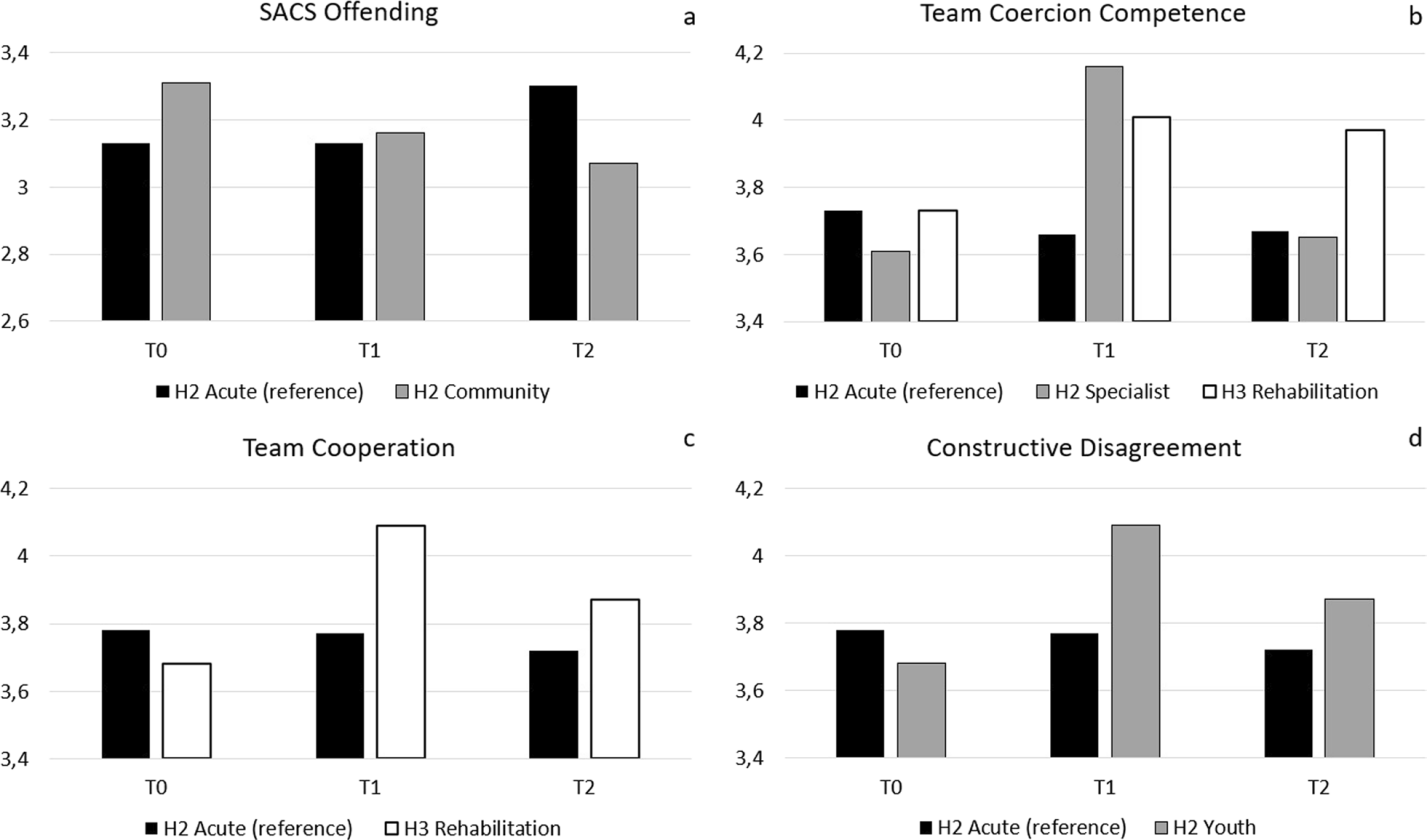
Statistically significant (*p* < 0.05) differences between Departments across T0, T1 and T2 in mean SACS Offending (panel a), Team Coercion Competence (panel b), Team Cooperation (panel c) and Constructive Disagreement (panel d)

Whereas in Hospital 2 Acute Care (the reference department) SACS **Offending** was higher at T2 than at T0 (b = 0.17, *p* < 0.05), the negative interaction effect for Hospital 2 Community Care showed that in this department it was lower at T2 than at T0, and that the difference was statistically significant (*b* = -0.41, *p* < 0.01; Fig. 1, panel a). **Team Coercion Competence** was 0.07 lower at T1 than at T0 in the reference department, yet interaction effects showed that in Hospital 2 Specialist it was 0.55 higher at T1 (i.e., −0.07 + 0.62) and that this difference was statistically significant (*p* < 0.01). The same applied to Hospital 3 Rehabilitation, where Team Coercion Competence was 0.28 higher at T1 (i.e., −0.07 + 0.35; *p* < 0.05; Fig. 1, panel b). **Team Cooperation** did not differ between T1 and T0 in the reference department (*b* = −0.01, *p* > 0.05), but in Hospital 3 Rehabilitation it was *b* = 0.41 higher at T1 (i.e., −0.01 + 0.42; *p* < 0.01; Fig. 1, panel c). **Constructive Disagreement** was 0.06 lower at T2 than at T0 in the reference department, while it was 0.31 higher (i.e., −0.06 + 0.37) in Hospital 2 Youth (*p* < 0.01; Fig. 1, panel d). No other statistically significant differences between Departments were found.

#### *Professions* (with ‘psychiatrists and related medical professions’ as reference group; see Fig. 2)

**Fig. 2:**
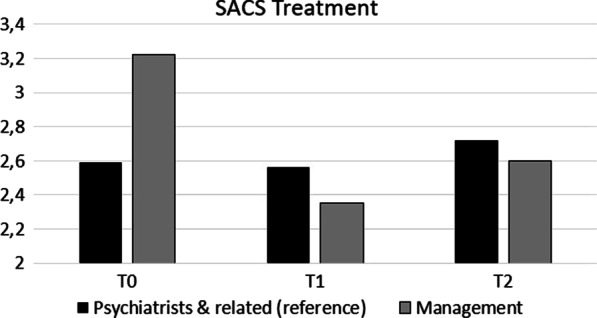
Statistically significant (*p* < 0.05) difference between Professions across T0, T1 and T2 in mean SACS Treatment

For professions, we found one statistically significant interaction effect. Specifically, compared to T0, SACS **Treatment** was 0.03 lower at T1 and 0.13 higher at T2 in the reference group (psychiatrists and related professions). In managers, it was 0.87 lower at T1 (i.e., -0.03–0.84; p < 0.01) and 0.62 lower at T2 (0.13–0.75; *p* < 0.001; Fig. 2) than at T0.

### Associations between ERG participation, case presentation and (changes in) outcomes

**Fig. 3:**
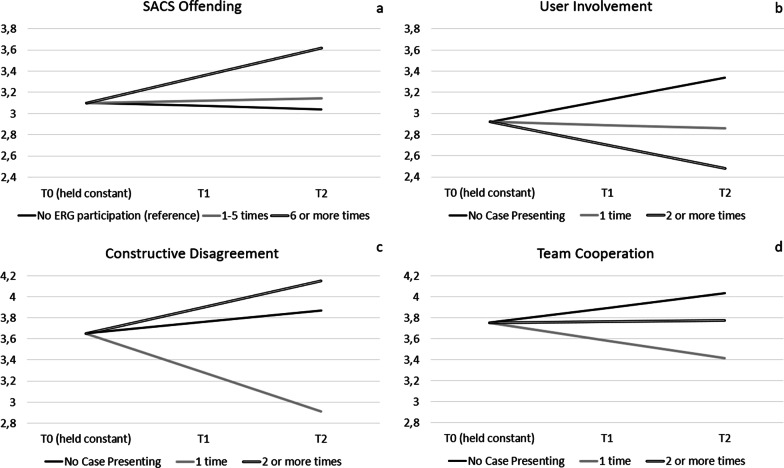
Statistically significant (*p* < 0.05) differences between **ERG Participation** groups in changes in SACS Offending (panel a) and between **Case Presenting** groups in changes in User Involvement (panel b), Constructive Disagreement (panel c) and Team Cooperation (panel d). Based on respondents who participated in at least two time points, had valid data on ERG Participation or Case Presenting, and baseline data for the outcome (for ERG participation: N = 160, for Case Presenting: N = 109). Models were adjusted for the baseline level of the outcome in order to make the initial outcome comparable between ERG and Case Presenting participants and non-participants

The models described in this section are based on participants with longitudinal data on the outcomes only, and with valid data on ERG participation (n = 160) and case presenting (n = 109), adjusted for department, profession, and baseline outcome (see Table 4 and Fig. 3). Because we were interested in mean changes over time on the individual level, we modelled time as a continuous variable, representing yearly change in outcomes.

**Table 4:** associations between ERG participation (n = 160) and case presenting (n = 109) and the outcomes in those who responded multiple times, adjusted for time, department and profession

Variable	SACS offending	SACS Care/Security	SACS Treatment	Team Coercion Competence	User Involvement	Team Cooperation	Constructive Disagreement
	B adj.^a)^	B adj.^a)^	B adj.^a)^	B adj.^a)^	B adj.^a)^	B adj.^a)^	B adj.^a)^
**# ERG participation** (ref = 0)							
Intercept(mean in ref)	3.10	4.03	2.51	3.70	2.89	3.73	3.63
1–5 times	−0.07	−0.08	−0.05	0.15*	0.19 ~	0.02	−0.06
6 + times	0.02	0.01	0.08	0.02	0.10	0.07	−0.05
**# Case presenting** (ref = 0)							
Intercept (mean in ref)	3.06	3.98	2.53	3.69	3.06	3.73	3.61
1 time	0.05	0.22*	0.01	0.17 ~	0.07	0.03	−0.02
2 + times	0.02	−0.04	−0.02	0.13	−0.11	0.06	0.02

a) Adj. = Adjusted for department, profession, and baseline outcome. Intercepts are unadjusted

~ *p* < 0.1; *p < 0.05; **p < 0.01; ***p < 0.001

The model without interaction effects with time (Table 4) showed that, compared to *not* participating in ERG, participating 1–5 times in ERG was associated with slightly higher reported **Team Coercion Competence** (*b* = 0.15, *p* < 0.05). In other words, respondents were slightly more positive about the competency of the team regarding the handling of coercion. Furthermore, compared to not presenting a case, presenting a case once during an ERG session was associated with higher reported SACS **Care & Security** (*b* = 0.22, *p* < 0.05; Table 4). In other words, case presenters perceived the use of coercion a bit more as Care & Security compared to those who did not present a case in an ERG session.

The model including interaction effects with time (Table 5) showed that whereas SACS **Offending** decreased by 0.03 per year in those *not* participating in ERG (*p* > 0.05), it increased by 0.26 (i.e. −0.03 + 0.29) per year in those participating 6 or more times per year (*p* < 0.05; Fig. 3, panel a). For case presenting, we found three significant interaction effects. First, whereas **User Involvement** increased by 0.21 per year in those who did *not* present a case (*p* < 0.001), it decreased by 0.22 per year (i.e., 0.21–0.43 =) in those who presented a case twice or more per year (*p* < 0.05, Fig. 3 panel b). Second, **Constructive Disagreement** increased by 0.11 per year in those who did not present a case (*p* < 0.05), whereas it decreased by 0.37 (i.e., 0.11–0.48) in those who presented a case once in a year (*p* < 0.01, Fig. 3 panel c). Lastly, whereas **Team Cooperation** increased by 0.14 per year in those who did *not* present a case (*p* < 0.01), it decreased by 0.17 per year (i.e., 0.14–0.31) in those who presented a case once (*p* < 0.05, Fig. 3 panel d).

**Table 5:** Interaction effects between time and ERG participation (n = 160) and time and case presenting (n = 109) on the outcomes in those who responded multiple times, adjusted for baseline outcomes, department and profession

Variable	SACS offending	SACS Care/Security	SACS Treatment	Team Coercion Competence	User Involvement	Team Cooperation	Constructive Disagreement
	B adj.^a)^	B adj.^a)^	B adj.^a)^	B adj.^a)^	B adj.^a)^	B adj.^a)^	B adj.^a)^
**# ERG participation** (ref = 0)							
Overall mean T0	3.10	4.07	2.53	3.71	2.92	3.75	3.65
Main effect time (in ref)	−0.03	−0.07	−0.04	0.01	−0.01	0.02	0.07 ~
1–5 times*Time	0.05	−0.11	0.09	0.03	0.06	−0.06	−0.06
6 + times*Time	0.29*	0.13	0.12	0.02	−0.13	−0.14	−0.13
**# Case presenting** (ref = 0)							
Overall mean T0	3.10	4.07	2.53	3.71	2.92	3.75	3.65
Main effect time (in ref)	−0.03	−0.07 ~	−0.03	0.10*	0.21***	0.14**	0.11*
1 time*Time	0.14	−0.10	0.15	−0.20	−0.24	−0.31*	−0.48**
2 + times*Time	0.21	−0.05	−0.04	0.07	−0.43*	−0.13	0.14

a) Adj. = Adjusted for department, profession and baseline outcome. Intercepts are unadjusted

~ *p* < 0.1; *p < 0.05; **p < 0.01; ***p < 0.001

## Discussion

This paper presents the results of a unique clinical ethics support evaluation study. By implementing structural Ethics Reflection Group (ERG) sessions (or Moral Case Deliberations; MCD) about the use of coercion at seven departments within three different Norwegian mental health care institutions, we studied variations in survey scores at three different time points within two years. In order to do so, we used panel data in a longitudinal design study at baseline, after 12 and after 24 months of implementing ERGs (T0-T1-T2).

This paper has shown that quantitatively measuring the impact of interventions is complex [85], and furthermore, that ERG or MCD should be perceived as complex interventions. The functioning and value of ERG or MCD depends on many things (e.g. the case at hand, the group dynamics, the facilitator and the way they are trained, the conversation method used, and the context in which ERG/MCD is implemented). As Schildmann and colleagues wrote, it is not at all clear which specific ingredient of ERG or MCD contributes to which specific impact [43]. Therefore, the results and the interpretation of the results of this study should be interpreted with caution. In what follows, we will briefly reflect upon the findings and then discuss some lessons learned with respect to interpreting and measuring the impact of ethics support and changes over time.

### Main results regarding variation at three time points and interpretation of results

In the multivariate analyses, taking all predictors into account, we found that the extent to which all respondents agreed that coercion can be seen as Care and Security decreased over time, possibly indicating a more critical attitude towards the use of coercion. Critical reflections and the sharing of doubts about the justification of coercion perhaps made participants respond in a more nuanced way towards the Care and Security items (see Additional file 1: Textbox 1). We also found that the extent to which respondents reported that they involved patients and families increased over time. Perhaps this can be explained by the fact that, during ERG and MCD, participants are specifically urged to consider patients’ and family’s viewpoints on coercion and related values and norms.

#### Results and interpretation of the results for departments

Within Community Care, we observed a significant decrease of seeing coercion as Offending. A possible explanation for the decrease may be that some health care professionals in community care felt that waiting too long before using coercion (for example since Norwegian law does not allow the use of coercion outside the hospital) might also cause harm or that, after ethical reflection about *how* to use coercion, professionals learned that coercion can be performed in a less offending way. Furthermore, respondents from both Rehabilitation and Specialist care perceived a better Team Coercion Competence. An explanation could be that for both departments, although they offer quite different settings, the joint team reflections about coercion cases made them aware that their competence regarding dealing with coercion increased during, and because of, the ERG sessions.

#### Results and interpretation of the results for Professions

We found only one significant difference among professions when looking at variation between the three time points. When compared with the group of ‘psychiatrists and related medical professions’, managers scored significantly lower in seeing the use of coercion as a possible Treatment; they started to slightly disagree with this view, while at T0 they were in doubt whether to coercion can be seen as a treatment. Managers are more distanced from the actual context in which coercion is used. Perhaps, through the participation in the ERG sessions or due to the extra focus on coercion during the two years of ERG implementation, managers became more critical about justifying the use of coercion as a treatment.

#### Results and interpretation of the results specifically related to participation in ERG

For participation in ERG, we found one significant change over time within the seven outcome parameters: those who participated in ERG six or more times each year perceived coercion clearly more strongly as Offending. Repeated ethical reflection groups about the use of coercive measures may have made these respondents more aware of the potential offending character of coercion and possible alternatives for the use of coercion.

#### Results and interpretation of the results specifically related to presenting a case in ERG

Those who presented their case in ERG more than 2 times a year gave lower scores for User Involvement. Perhaps, due to the ERG sessions, they started to realize that they knew relatively little about what patients’ and families’ specific values, norms and perspectives are with respect to the use of coercion. Interestingly, those who presented a case in ERG once a year gave lower scores for Constructive Disagreement and for Team Cooperation than those who did not present a case. One possible explanation is that positive experiences with case presenting in ERG made case presenters realize that usually, at the unit, the team cooperation and handling of disagreement do not happen in the same positive way as during the ERG sessions. However, this does not explain why those who presented a case more often did not show the same significant change in scores. At the same time, ERG and MCD are often used for strengthening team cooperation and dealing more constructively with disagreement [2, 10, 18, 21, 23, 25] and several qualitative evaluation studies confirm the achievement of these goals through ERG.

We found more significant changes over time for the other parameters due to Participation in ERG and Case presentation in ERG, yet they did not remain statistically significant after adjustment for departments and professions within the statistical analyses. Perhaps the departments already had very different points of departure concerning their normative attitudes regarding coercion and user involvement, including different cultures for team cooperation and the handling of disagreement. Furthermore, the number of different professions participating in training and courses on the use of coercion might vary among departments. Future CES evaluation research should focus in more detail on the specific characteristics of the involved departments and professions in order to better understand their possible contribution to changes over time when implementing CES.

### Overall interpretation of changes over time: Response shift and normative evaluation

Above, we described that not only studying ERG as an intervention and evaluating changes over time are complex matters; interpretating changes over time in respondents’ answers can also be complex. Changes over time may be explained by the fact that the phenomena under study (i.e. the outcome parameters) actually changed during the time of this study. Yet, they may also be explained by various kinds of ‘response shift’. ‘Response shift’ was defined by Sprangers and Schwartz [82] as a change in the meaning of the self-evaluation of a target construct. Response shift can be caused by (a) a redefinition of the target construct (i.e. reconceptualization of what coercion actually means or how one should interpret ‘Offending’); (b) a change in the respondent’s values (i.e. reprioritization of importance of domains substituting the target construct); or (c) a change in the respondent’s internal standards of measurement (scale recalibration). There are possibilities to check and calculate whether there is a response shift, but because there were few longitudinal data in this study, this was not possible here [85; see 8.5.6].

Another precaution concerns the way in which changes over time can be interpreted *normatively*. This of course applies to drawing normative conclusions based on empirical results in general [84]. However, this certainly applies to research where the aim is to study changes in normative attitudes after ethics support interventions such as ERG or MCD sessions. Drawing normative conclusions, e.g. whether a specific result or outcome can be interpreted as morally better or as a moral improvement is a complex matter [35]. For example, given the initial hypotheses of this study, it sounds perhaps plausible that seeing coercion as more offending, after two years of critical reflection on moral challenges regarding coercion, could be seen as a desirable and hence morally good result. Yet, after deliberation in ERG, and discovering ways of performing coercion in a more transparent and respectful way, respondents perhaps also realized that coercion can be performed in a less offending way. In order to draw normative conclusions when interpreting the results of this study, complementary qualitative data are needed, e.g. thick descriptions of specific situations in which employees use coercion. Researchers can then study these together with respondents in order to discover how to interpret and judge the specific situation. Finally, as mentioned in the Background section, one should not automatically conclude that positive outcomes of CES will eventually become the primary goal of or justification for CES. Stimulating ethics reflection by means of implementing ERGs or MCDs has value in itself. Despite the value and importance of CES evaluation studies in general, participating in ERGs and MCDs should not become instrumentalized as an intervention in which the only aim is to reach specific outcomes. This would threaten the inherent intellectual and normative freedom of ethics reflection within ERG and MCD.

### Relationship with other ERG or MCD impact evaluation studies

This study took place within a much larger study, in which qualitative analyses of transcribed focus groups about experienced changes over time were also used [54]. Focus group respondents reported that they improved their professional competence and confidence, developed greater trust within the team, and experienced more constructive disagreement and room for internal critique (i.e. fewer judgmental reactions and more reasoned approaches) [54]. This resembles some of the changes shown in the Constructive Disagreement scale within this paper but this is not confirmed by changes in the Team Cooperation scale in this paper.

Several results from other ERG and MCD evaluation studies, which focused explicitly on the outcomes and changes after a series of ERG or MCD, resemble the results described in this paper [2, 10, 18, 21, 25]. In a recent systematic literature review in which 25 empirical papers on the quantitative and qualitative evaluation of ERG or MCD were analysed to identify various impacts of ERG or MCD, Haan and colleagues found a change in professional opinion or attitude and a more critical attitude towards professionals’ practice [23]. This relates to our findings, where respondents became more aware of and more critical towards the use of coercion. Haan and colleagues also mentioned that several studies found that ERG or MCD reduces conflicts and leads to more solidarity, respect, tolerance, collegial support and cooperation. Again, these findings resemble some of the changes in Constructive Disagreement found in our study. However, as mentioned above, one should remain careful in suggesting a linear causal relationship between interventions such as ERG or MCD and reported or observed changes over time. Furthermore, Haan et al. reported that MCD participants were more aware of patients’ and families’ rights in the decision-making process and more often considered the patients’ and families’ perspectives, wishes and needs. This is in line with the significant increases for User Involvement in this study. Finally, Haan et al. concluded that empirical evidence of ERGs or MCDs concrete impact on the (improvement of the) quality of patient care is limited and mostly based on self-reports [23]. This clearly sets the agenda for future CES evaluation studies: to study in more detail the actual impact of CES on the quality of care.

### Strengths and limitations of the study

A unique strength is the fact that this study focuses on the variation of measures at three time points within two years of ERG (or MCD). We are not aware of similar studies carried out before. Furthermore, instead of asking participants directly how they perceived changes over time at T1 and T2, we used the same factual and normative statements at three time points. A strength is also the fact that all ERG or MCD facilitators received the same amount of training (5 days) and used the same conversation method for ERG or MCD (i.e. the CME model [10]). Another strength is that this study combines a specific clinically and ethically relevant topic, i.e. the use of coercion in mental health care, with more general evaluative measures of clinical ethics support (CES), such as normative attitudes, team cooperation and constructive disagreement. The latter three categories for outcome parameters fit well with what the intervention ERG or MCD is supposed to do. Finally, this study provided worthwhile insights in how to develop and execute this specific research design and used methodology which future CES evaluation researchers might benefit from (see paragraph ‘Recommendations’ below).

An important limitation of this study is the small amount of longitudinal data. This stresses the importance of guiding and monitoring the response rate more intensive in future CES evaluation studies. The linear mixed model analyses helped us in this respect; although they form a well-known statistical procedure [81], more longitudinal data is preferable to create stronger validity of the results. Furthermore, studying variation in scores for different departments in different hospitals made it difficult to relate the variations in scores to the ERG or MCD sessions themselves, since the culture of the departments, the amount of coercion used, and the type of coercion used may vary. In addition, it is not clear what outcomes would be clinically and practically relevant; therefore, we could not calculate whether the statistical power for this study was adequate. Another limitation is the fact that despite the significant variation in scores at the three time points, the differences between no, little or much ERG participation were generally small in absolute terms. More in general, future studies should be more explicit about whether ‘meaningful changes’ might refer to useful changes in the light of trying to measure change after a complex intervention (i.e. from the viewpoint of the research aim) OR to clinically relevant changes. A meagre comfort is perhaps that, when measuring variation at different time points after the implementation of a complex intervention, serious methodological challenges almost always arise [43]. According to Craig et al. [85], a lack of demonstrable effects of any complex intervention may perhaps rather reflect implementation and methodological challenges rather than the actual ineffectiveness of the intervention. A final limitation is the fact that we made use of self-developed scales, except for the validated SACS scales. These self-developed scales were piloted and had reliability scores varying from Cronbach’s α 0.62 for Constructive Disagreement to 0.83 for Team Cooperation, but they were not validated. The self-developed scales should therefore be used for further validation in the field of CES evaluation studies. Furthermore, besides the use of scales for measuring respondents’ attitudes and perceptions, the use of objective outcomes such as specific events can be helpful, e.g. the frequency and duration of use of coercive measures.

### Recommendations for future ethics support evaluation research

This is an innovative study when it comes to measuring intervention-specific outcome parameters for describing the impact of clinical ethics support (i.e. ERG or MCD). Experiences with this kind of explorative studies on the impact of CES might pave the way to new mixed-method study designs with control groups (e.g. stepped-wedge design) and some sort of randomization in combination with the use of qualitative research methods (e.g. interviews and focus groups). In the selection of departments, groups or teams, it is important that, prior to the start of the study, one takes into account the core professional tasks and/or the specific team cultures, e.g. related to dealing with moral doubts, hierarchy, mutual exchange of feedback and the presence or lack of a safe atmosphere. These could become confounders for the specific ethics support intervention. It can be useful to use specific baseline measurements to get an indication of the specific differences, e.g. ethics climate and team cooperation scales. With respect to the specific ethics support intervention, one should try to develop the same kind of procedures for the process of the ethics support intervention (e.g. the same training for all ERG facilitators and the same conversation method). More or stronger significant changes may result from measuring impact in relatively small teams or units as well as participants’ relatively high frequency of participation in the ERG or MCD sessions. In order to increase the response rate, the presence of the researchers at the study site and a clear explanation of the potential value of this study may be helpful. The researchers’ presence will also make it easier to link identical participants with subsequent questionnaires in order to increase the amount of longitudinal data. Finally, it is important to use validated measures for CES outcomes and types of outcomes that fit the specific ingredients of the particular CES intervention (e.g. the European questionnaire for measuring outcomes of MCD sessions (i.e. the EURO-MCD 2.0 [25]).

## Conclusions

This paper presents the research design, research methodology and results of a unique clinical ethics support evaluation study in which changes over time among health care professionals’ attitude and perceptions were measured after two years of Ethics Reflection Groups (ERG) or Moral Case Deliberations (MCD). Despite the little amount of longitudinal data, we found indications that structural ERGs or MCDs at their departments might contribute to employees reporting a more critical normative attitude towards coercion. We observed significant differences in outcomes among both Departments and Professions. Furthermore, participants who participated frequently in ERG sessions perceived the use of coercion as more Offending. Those who presented a case in the ERG sessions showed significantly lower scores on User Involvement, Team Cooperation and Constructive Disagreement. Initial significant changes due to frequency of Participation in ERG and Case presentation in ERG did not remain statistically significant after adjustment for Departments and Professions. Future CES evaluation research should therefore focus in more detail on the specific characteristics of the involved departments and professions. Since differences were generally small in absolute terms, we recommend further studies to shed more light on the clinical relevance of changed outcomes over time.

It is difficult yet important to study changes over time in clinical practice after the implementation of CES and to try and find a relationship between CES interventions and CES outcomes. This paper gives some suggestions for improving the design and validity of future CES evaluation research. This study is a first step to further construct and adjust scales for CES evaluation studies. It is crucial to learn about how clinical ethics support contributes to team cooperation, the handling of disagreements and the quality of care—for researchers, for health care professionals, for ethics support staff, and, last but not least, for patients. Indeed, despite the intrinsic value of participating in ethics support activities such as ERG or MCD, clinical ethics support inherently aims, and should aim, at improving clinical practices.

## Supplementary Information

**Additional file 1**. **Appendices**. **Textbox 1**: The 15 normative statements of the SCAS with three subscales. **Textbox 2**: The 6 statements about the competence of the team regarding use of coercion. **Textbox 3**: The 11 statements about involvement of patients and family in situations of coercion. **Textbox 4**: The 13 statements about team cooperation. **Textbox 5**: The 8 statements about constructive disagreement.

## Data Availability

The datasets used and/or analysed during the current study are available from the corresponding author on reasonable request.

## Footnotes

1 MCD and ERG are synonyms for the same activity: a structured case discussion on a real case within a group, facilitated by a trained facilitator. From now on we will use the term ERG only for ease of reading.

2 Some examples of the variety of research questions within CES evaluation studies are: How is the CES organised and implemented?; How and how often is the CES executed?; What kind of ethical issues are discussed during CES?; What kind of outcomes do CES participants experience and how important are they?; How did the patient and family participate during the CES?; What is the quality of the CES service?; What is the quality of the deliberation within CES?; How to write reports of CES?

3 ERG can have many different goals. Usually, authors distinguish the following levels or domains of goals of ERG: (a) case-related goals (e.g. finding alternative actions); (b) goals related to the empowering of professionals’ moral competency; (c) goals related to improving multidisciplinary cooperation; and (d) goals related to developing policy or organisational change [12, 14, 44, 45]. Hence, CES evaluation studies focusing on outcomes should make explicit which goals are at stake for the specific kind of CES.

4 For example, when one focus on less medical consumption as a CES outcome. According to a review of RCTs for CES evaluation, Chen and Chen [46] found three papers based on two RCT studies in the USA on the evaluation of CES outcomes. In those studies, Schneiderman et al. [47, 48] and Gilmer et al. [49] found: “For patients who did not survive to hospital discharge, ethics consultations were significantly associated with shorter ICU stays, shorter hospital stays, less use of life-sustaining treatments and lower hospital costs” [47; p. 595). Although these are interesting and somehow plausible results (like the results of a more recent Asian study [50]), it could become morally problematic if these outcomes (i.e. reducing consumption of medical resources) become one of the major aims of CES.

5 This PET study, which took place from 2011 until 2016, included four sub-studies: (a) a systematic literature review on the evaluation of ethics support in mental health care [18], (b) interviewing patients, their children and other family about coercion and involvement [56, 71, 72], (c) the implementation and evaluation of ERG [4, 18, 54] including an enumeration of ethical challenges related to coercion [73, 74], and (d) a national survey among mental health care staff and patients on normative attitudes related towards coercion [75, 76]. The results presented in this paper are from part c of the PET study. For all PET-related papers, see https://www.med.uio.no/helsam/forskning/prosjekter/pet/publikasjoner/index.html

6 Some of these ethicists or trainers were also researchers in the PET study.

7 The reliability of all the seven scales was satisfactory within this study, varying from 0.62 Cronbach’s α for Constructive Disagreement to 0.83 Cronbach’s α for Team Cooperation.

8 ‘Factual’ statements are statements about how respondents perceive the facts regarding a specific phenomenon (e.g. the way employees involve patients during the use of coercion). ‘Normative’ statements are statements about how respondents think or judge about a topic (e.g. ‘use of coercion is wrong’).

9 Bear in mind that these are repeated cross-sectional measurements in which only some ERG participants were included multiple times. Therefore, these averages do not directly demonstrate changes in individual attitudes during the study period.
