# Supplementary material for: Two years of ethics reflection groups about coercion in psychiatry. Measuring variation within employees’ normative attitudes, user involvement and the handling of disagreement
Source: BMC Med Ethics. 2023 May 12;24:29. doi: 10.1186/s12910-023-00909-w (PMC10182617; doi:10.1186/s12910-023-00909-w)
Supplement: Supplementary file 1 — Additional file 1. Appendices. Textbox 1: The 15 normative statements of the SCAS with three subscales. Textbox 2: The 6 statements about the competence of the team regarding use of coercion. Textbox 3: The 11 statements about involvement of patients and family in situations of coercion. Textbox 4: The 13 statements about team cooperation. Textbox 5: The 8 statements about constructive disagreement. [file 12910_2023_909_MOESM1_ESM.docx]

**SUPPLEMENTARY MATERIAL (17FEB2023)**

**RELATED TO**

**Title page**

Two years of ethics reflection groups about coercion in psychiatry. Measuring variation within employees’ normative attitudes, user involvement and the handling of disagreement

**Authors:** Bert Molewijk^1&2 *^, Reidar Pedersen^1^, Almar Kok^3&4^, Reidun Førde^1^, Olaf Aasland^1^

**APPENDICES**

**Textbox 1: The 15 normative statements of the SCAS with three subscales, Husum et al. [79]**

Coercion seen as offending (SACS I; 6 items; ‘offending’)

3. Use of coercion can harm the therapeutic relationship

4. Use of coercion is a declaration of failure on the part of the mental health services

8. Coercion violates the patient integrity

13. Too much coercion is used in treatment

14. Scarce resources lead to more use of coercion

15. Coercion could have been much reduced, when giving more time and personal contact

Coercion seen as needed for care and security (SACS II; 6 items; ‘care & security’)

5. Coercion may represent care and protection

2. For security reasons coercion must sometimes be used

1. Use of coercion is necessary as protection in dangerous situations

7. Coercion may prevent the development of a dangerous situation

11. Use of coercion is necessary towards dangerous and aggressive patients

9. For severely ill patients coercion may represent safety

Coercion seen as a treatment (SACS III; 3 items; ‘treatment’)

6. More coercion should be used in treatment

12. Regressive patient require use of coercion

10. Patients without insight require use of coercion

**Textbox 2: The 6 statements about the competence of the team regarding use of coercion**

1. We usually have enough qualified colleagues at our unit during one shift in order to take care for situations in which coercion occurs.
2. In general, our colleagues at our unit have enough competence about how to prevent using coercive measures.
3. In general, our colleagues at our unit have enough competence in order to perform/execute coercive measures in a good way.
4. Our colleagues receive support from the management when they try to prevent the use of coercion.
5. At our unit, we have good procedures and routines in order to prevent the use of coercion.
6. Our colleagues at our unit have enough knowledge about legislation and rules for the use of coercion.

**Textbox 3: The 11 statements about involvement of patients and family in situations of coercion**

1. We discuss with the patient if and how they experienced coercion before.
2. Patients are informed about how coercion will be executed, before we are in the actual situation in which coercion might be necessary.
3. Before we are in the actual situation in which coercion might be necessary, we ask the patient what kind of alternatives for coercion we should try.
4. When we use coercion, we ask the patient beforehand what kind of coercive measure he/she prefers.
5. When the use of the coercive measure ends, we ask the patient how he experienced the use of the coercive measure.
6. When the use of the coercive measure ends, we ask the patient what we could do to prevent the use of the coercive measure in the future.
7. We inform most near relatives how we execute coercion, before we are in the actual situation in which coercion might be necessary.
8. Before the coercion is executed, we ask the most near relatives which alternatives for the use of coercion we should try.
9. We ask most near relatives about their viewpoints during the execution of coercion.
10. During the use coercion, we ask the patient about alternative solutions for coercion (in order to reduce the duration of the coercion).
11. After the use of coercion, we discuss the future prevention of coercion with most near relatives if the patient consents for that or if the patient misses capacity to consent.

**Textbox 4: The 13 statements about team cooperation** (from Schippers et al. [80] & Kalvemark Sporrong et al. [81])

We talk about different ways in which we can reach our objectives. (item 5 from Schippers)

We work out what we can learn from past activities. (9 Schippers)

- During task execution, we seldom stop to assess whether the team is on the right track. (11 Schippers)

We check whether our activities produced the expected results. (18 Schippers)

- At this unit we do not evaluate the way we cooperate. (2 Schippers)

If things don't work out as planned, we consider what we can do about it. (22 Schippers)

- The methods used by the team at my unit to get the job done are seldom discussed. (27 Schippers)

We regularly discuss whether the team at my unit is working effectively. (28 Schippers)

Problems which should get solved are often looked at from different points of view at my unit. (15 Schippers)

- Those with whom I work have little understanding about how I think regarding difficult decisions. (Kalvemark Sporrong)

At my unit, different values and opinions tolerated. (Kalvemark Sporrong)

At my unit, we discuss together about ethical dilemmas. (Kalvemark Sporrong)

Before we get to solve our tasks together, we make sure everyone in the team at my unit has the same understanding of what the problem is. (10 Schippers)

**Textbox 5: The 8 statements about constructive disagreement** (partly from Kellermanns et al. [82])

1. At my unit, there is a useful give‐ and take

2. At my unit, there is a constructive challenge of ideas, beliefs, and assumptions

3. - At my unit, it is dangerous to express disagreement and criticism (not from Kellermanns et al.)

4. At my unit, we are more concerned with different opinions or views focus on issues rather than on disagreement between persons (inspired from Kellermanns et al.)

5. At my unit, we respect each other’s viewpoint even if we disagree (inspired from Kellermanns et al.)

6. At my unit, the merits of new ideas are openly discussed

7. - At my unit, decisions are dominated by position and power

8. - At my unit, things are decided without the involvement of employees (not from Kellermanns et al.)
